# Genome-Wide Analysis of Trehalose-6-Phosphate Phosphatases (TPP) Gene Family in Potato (*Solanum tuberosum*) Reveals Functional Divergence Under Stress

**DOI:** 10.3390/plants14213300

**Published:** 2025-10-29

**Authors:** Shuwen Huang, Naiqian Li, Yi Yang, Anjing Wang, Caicai Lin, Peiyan Guan, Xia Zhang, Shuangshuang Zheng, Gang Zhang, Yufei Guo, Wenhui Guan, Sajidam Amat, Linshuang Hu, Qingshuai Chen

**Affiliations:** 1 Shandong Provincial Key Laboratory of Biophysics, Institute of Biophysics, Dezhou University, Dezhou 253023, China; 13280270966@163.com (S.H.); 2022110577@sdau.edu.cn (N.L.); yangyi@dzu.edu.cn (Y.Y.); anjingwang@dzu.edu.cn (A.W.); lcc@dzu.edu.cn (C.L.); zhangxia@dzu.edu.cn (X.Z.); zssadu@163.com (S.Z.); gzhang@dzu.edu.cn (G.Z.); 13356196241@163.com (Y.G.);; 2 Belgorod Institute of Food Science, Dezhou University, Dezhou 253023, China; 3 National Key Laboratory of Wheat Improvement, College of Life Sciences, Shandong Agricultural University, Tai’an 271018, China; 4 College of Life Science, Dezhou University, Dezhou 253023, China; peiyanguan@dzu.edu.cn

**Keywords:** trehalose-6-phosphate phosphatase (TPP), potato, genome-wide analysis, expression pattern, abiotic stress

## Abstract

Trehalose-6-phosphate phosphatase (TPP) modulates the Trehalose-6-phosphate–trehalose balance, a key regulatory node in plant carbon sensing and stress resilience. However, its functional roles in vegetative crops such as potato (*Solanum tuberosum*, *St*) remains poorly understood. Here, we conducted a genome-wide identification of the *StTPP* gene family and identified nine distinct loci distributed across five chromosomes. Phylogenetic analysis categorized these loci into three clades, supported by conserved HAD-box motifs and distinct exon–intron structures. Family expansion was driven by segmental duplication under purifying selection. In silico promoter analysis revealed cis-elements responsive to hormones, light, and stress, while network modeling identified 64 transcription factors potentially involved in regulating *StTPP* expression. A biphasic transcriptional response was observed in the salt-tolerant cultivar Xisen6: rapid induction of *StTPP2/3/9* early in salt exposure, followed by late repression of most members. Subcellular localization assays indicated that StTPP3 is present in the nucleus and cytosol, suggesting multifunctional roles. These findings suggest that StTPPs integrate developmental and environmental signals, providing a molecular basis for improving potato stress tolerance and yield stability.

## 1. Introduction

Amid accelerating climate change, ensuring global food security has emerged as one of the most urgent challenges of the 21st century [1]. *Solanum tuberosum* L., commonly known as potato, is the fourth most cultivated food crop globally, following wheat (*Triticum sativum* L.), rice (*Oryza sativa* L.), and maize (*Zea mays* L.), with cultivation spanning over 19 million hectares. Potato serves as a primary carbohydrate source for more than 1 billion people [2]. However, its yield is highly susceptible to abiotic stresses. For example, drought during the critical tuber formation phase can reduce yields by up to 50% [3]. Intermittent heatwaves and salinity shocks also hinder tuber growth and quality [4,5]. Climate projections indicate that the frequency of such stress events will increase in major potato-growing regions, exacerbating the gap between global demand and supply constrained by climate factors [6]. Consequently, understanding the molecular networks underlying potato’s stress responses and identifying genetic targets for resilience breeding have become critical agricultural priorities.

Trehalose metabolism has emerged as a conserved and potent regulator of plant stress tolerance [7]. Trehalose (α-d-glucopyranosyl-(1,1)-α-d-glucopyranose), a non-reducing disaccharide found ubiquitously across prokaryotes and eukaryotes, functions both as a compatible osmolyte protecting membranes and proteins during dehydration and as a signaling molecule regulating carbon allocation, growth, and defense responses [8,9]. Central to this regulatory system is trehalose-6-phosphate (Tre6P), an intermediate that serves as a sucrose-status sensor by inhibiting the energy sensor sucrose non-fermenting-1-related protein kinase 1 (SnRK1) and integrating abscisic acid (ABA) signaling [10,11,12,13]. The cellular concentration of Tre6P in plants is tightly regulated by two opposing enzymes: Tre6P synthase (TPS), which catalyzes Tre6P formation, and Tre6P phosphatase (TPP), which de-phosphorylates Tre6P to form trehalose, thus maintaining metabolic equilibrium [14].

Accumulating evidence demonstrates that modulating TPP expression or activity can simultaneously enhance abiotic stress tolerance and sustain yield under adverse conditions by fine-tuning sugar signaling and carbon partitioning [15,16,17,18,19,20]. For instance, elevated Tre6P levels correlate with improved drought resilience in maize and increased grain filling in rice [18,21], highlighting its dual role as a metabolic integrator and agronomic trait modulator.

Plant TPP proteins belong to the L-2-haloacid dehalogenase (HAD) superfamily and contain a conserved phosphatase box (DxDxT/V) essential for catalysis [22]. Despite this structural conservation, *TPP* genes exhibit considerable functional diversity. They vary in subcellular localization (cytosol, chloroplast, or nucleus), catalytic efficiency, and spatio-temporal expression patterns [23]. Gene family expansions via tandem and segmental duplications have contributed to sub-functionalization and neo-functionalization events across angiosperms [24]. Functional studies in diverse species highlight the physiological versatility of TPP enzymes. In Arabidopsis, chloroplast-localized AtTPPD mediates redox-dependent salt tolerance [25], while rice endosperm-restricted OsTPP7 accelerates coleoptile elongation under anaerobic germination [26]. In maize, ZmTPP10 (RAMOSA3, RA3) accumulates specifically in young inflorescences and influences grain yield by modulating axillary meristem fate [27,28]. These examples illustrate how regulatory and expression divergence among TPP paralogs enables specialized physiological outputs, reinforcing the need for crop-specific characterization to identify optimal candidates for breeding.

Potato, a globally vital, vegetatively propagated tetraploid (2n = 4x = 48) with high heterozygosity, is particularly sensitive to transient drought, salinity, and heat stress during the stolon-to-tuber transition—a critical window when sucrose unloading and starch biosynthesis are tightly coordinated by metabolic signals such as Tre6P [29]. Although eight *TPS* genes have been functionally annotated in potato [30], the *TPP* gene family remains uncharacterized at the genome-wide level, representing a major gap in our understanding of Tre6P-mediated regulation in this crop. The availability of the high-quality DM1-3 v6.1 reference genome [31,32] and extensive transcriptomic resources now enables systematic identification and functional inference of *StTPP* genes.

This study presents the first comprehensive genome-wide analysis of the potato *TPP* gene family. Nine putative *StTPP* loci were identified, classified, and characterized using phylogenetic, syntenic, and protein motif analyses to trace their evolutionary origins. In silico promoter profiling identified hormone- and stress-responsive cis-elements, as well as potential transcription factor (TF)-DNA regulation networks. Meta-analysis of public RNA-seq datasets, along with real-time fluorescence quantitative polymerase chain reaction (RT-qPCR) assays, revealed tissue-specific and abiotic stress-responsive expression patterns under drought, salinity, heat, and cold. Potato encodes a Solanaceae-expanded *StTPP* repertoire with divergent regulatory motifs and stress-inducible expression. Specific *StTPP* members are likely coordinately induced in roots and tubers under abiotic stress to fine-tune Tre6P homeostasis and carbon partitioning. The candidate *StTPP* genes identified in this study will facilitate functional investigations and provide molecular targets for genome editing or marker-assisted selection in the development of climate-resilient potato cultivars.

## 2. Results

### 2.1. Identification and Analysis StTPP Family in Potato

**Figure 1:**
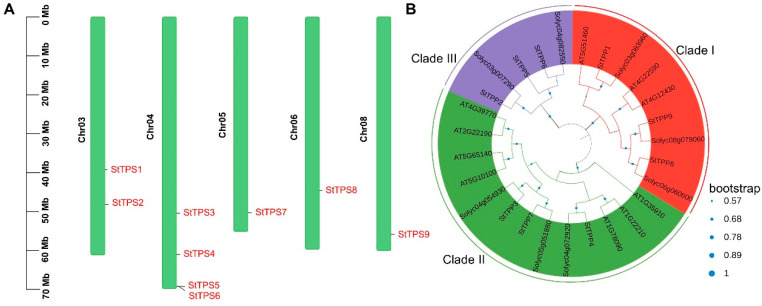
Genomic distribution and phylogenetic classification of *StTPP* genes. (**A**) Chromosomal mapping of StTPP loci in potato. Chromosome numbers are shown on the left, with corresponding gene positions aligned to the right. Scale bars indicate relative chromosomal lengths. (**B**) Phylogenetic analysis of TPP proteins from (*Solanum tuberosum*, St), Arabidopsis (*Arabidopsis thaliana*, At), and tomato (*Solanum lycopersicum*, Sl), inferred by the maximum likelihood method using the Jones–Taylor–Thornton (JTT) model in MEGA7, based on full-length amino acid sequences. Bootstrap support values (from 500 replicates) are normalized and displayed as blue circles on the branch.

Genome-wide mining of the potato (*Solanum tuberosum* L. DM v6.1) assembly identified nine complete TPP loci, designated StTPP1-StTPP9 based on their sequential chromosomal locations (Table 1, Figure 1). The coding and amino acid sequences are provided in Table S1. Physicochemical profiling revealed that mature StTPP peptides range from 289 (StTPP5) to 386 (StTPP3) residues, with predicted molecular masses ranging from 32.84 kDa (StTPP5) to 43.48 kDa (StTPP3). The isoelectric points (pI) ranged from acidic pI 5.44 (StTPP2) to basic pI 9.45 (StTPP4). Grand average hydropathicity (GRAVY) values indicated that all StTPP proteins are hydrophilic, ranging from −0.512 (StTPP4) to −0.194 (StTPP5). These data provide a comprehensive inventory and essential biochemical descriptors for each StTPP member in potato, serving as a reference for subsequent functional studies.

### 2.2. Chromosomal Distribution and Phylogenetic Analysis of StTPP Genes in Potato

Chromosomal mapping revealed that the nine StTPP loci are distributed across only five of the twelve potato chromosomes. A significant cluster of four genes is located on chromosome 4, with two copies on chromosome 3. Chromosomes 5, 6, and 8 each contain a single representative locus. No StTPP sequences were found on chromosomes 1, 2, 7, 9, 10, 11, or 12 (Figure 1A), suggesting a non-random genomic distribution that may reflect a lineage-specific evolutionary history.

A maximum-likelihood phylogeny, constructed from 27 full-length TPP proteins, nine from potato (StTPP), eight from tomato, and ten from Arabidopsis, revealed three monophyletic clades (I–III; Figure 1B). Potato contributed three members to each clade, indicating balanced retention following the Solanum–Arabidopsis split. Within clade III, StTPP2/5/6 formed a well-supported lineage, which is sister to but topologically distinct from the corresponding Arabidopsis subfamily. In contrast, clades I and II retained the same internal branching observed in Arabidopsis, a pattern also documented in wheat, tomato, and soybean [19,33,34]. These conserved topologies validate the use of Arabidopsis functional data to predict StTPP activity, while the divergence in clade II highlights potato-specific evolutionary paths.

### 2.3. Conserved Motif, Domain, and Gene Structure Analysis of StTPP

**Figure 2:**
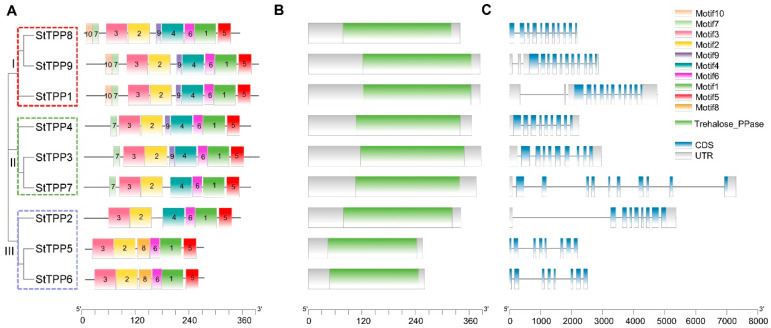
Integrative annotation of StTPP proteins and loci. (**A**) Maximum-likelihood phylogenetic tree of nine full-length potato TPP sequences, categorized into three distinct, well-supported clades (I–III, color-coded). Evolutionary distances are represented by branch lengths. MEME (v5.5.2) identified ten conserved motifs (color blocks) common to StTPPs. (**B**) Conserved domain architecture predicted by Pfam, showing the Trehalose_PPase catalytic domain (Pfam PF02358) as green rectangles. (**C**) Gene structure schematic of *StTPP* loci, with coding sequences in lake blue, introns in black, and 5′/3′ UTRs in gray. Scale bars denote amino acid length (**A**,**B**) and genomic span (**C**).

Multiple Expectation Maximization for Motif Elicitation (MEME) analysis identified ten non-redundant motifs across the nine StTPP proteins (Figure 2A), with the conserved sequences for motifs 1–10 provided in Figure S1. Motif distribution was subgroup-specific: clade I members possess nine motifs, excluding motif 8, while clades II and III retain six to seven motifs in a consistent order. This motif consistency supports the phylogenetic classification.

Pfam screening confirmed the presence of a single Trehalose_PPase domain (PF02358) in each StTPP protein, classifying them within the HAD superfamily [22] (Figure 2B). To assess evolutionary conservation, we performed a multiple sequence alignment of all TPP proteins from potato, tomato, and Arabidopsis, together with the well-characterized maize RA3 protein as an outgroup. Four canonical catalytic motifs were identified across all species: Motif I [DxDx(T/V)], Motif II (S/T), Motif III (K), and Motif IV [(G/S)(D/S)xxx(D/N/E)] (where x denotes any amino acid) (Figure S2). Strikingly, Motifs I–III were completely conserved in all analyzed TPPs. In contrast, Motif IV was highly conserved in potato, tomato, and Arabidopsis, conforming to the consensus (e.g., GDDRTD in StTPP1), while in maize RA3, Motif IV diverged significantly, adopting the sequence GATTSS. This substitution aligns with prior reports that RA3 may function as a regulatory scaffold rather than a canonical enzyme [35]. These findings underscore that the core catalytic machinery of TPPs is preserved in potato and closely related species, while lineage-specific alterations—such as in maize RA3—may underlie functional diversification.

Alignment of genomic and coding sequences revealed exon counts ranging from eight to eleven and intron counts from seven to ten (Figure 2C). Splice patterns within clades are nearly identical, with clade I genes exhibiting identical exon–intron junctions. This architectural consistency supports the phylogenetic subgroups and suggests that intron–exon variations may contribute to functional divergence. Notably, *StTPP7* exhibits the largest genomic span, primarily due to its inclusion of multiple exceptionally long introns. Detailed sequence analysis of these introns revealed no significant enrichment of transposons or other repetitive sequences, indicating that their expansion was not driven by transposon insertion. Instead, these introns are predominantly composed of unique non-repetitive sequences with a high AT content, which may influence local chromatin conformation or splicing kinetics. Together, these observations indicate that *StTPP7* has undergone a unique evolutionary trajectory, potentially contributing to sub-functionalization within the *StTPP* family.

In summary, motif, domain, and gene-structure analyses converge to validate the three StTPP clades, establish a conserved catalytic framework, and suggest that structural divergence may drive functional specialization in potato.

### 2.4. Duplication History and Selective Constraints of StTPP Genes

**Figure 3:**
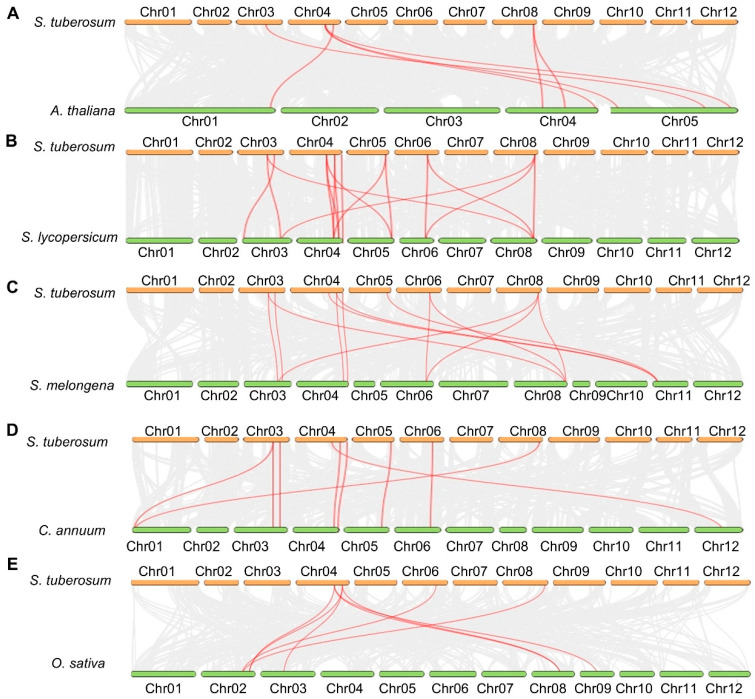
Comparative synteny of potato TPP genes across species. Whole-genome collinearity is illustrated between potato and Arabidopsis (**A**), tomato (**B**), eggplant (**C**), pepper (**D**), and rice (**E**). Gray ribbons represent syntenic blocks, while red ribbons mark orthologous TPP pairs, underscoring both evolutionary conservation and lineage-specific expansion of StTPP loci across dicot and monocot genomes.

The evolutionary history of the potato TPP family was reconstructed by integrating synteny data from Arabidopsis, five Solanaceae species (including potato, tomato, pepper, and eggplant), and rice (Figure 3 and Figure S3). Using the Multiple Collinearity Scan toolkit X version (MCScanX), we identified one tandem duplication event and four segmental duplication events. The pair *StTPP5* and *StTPP6* was classified as a tandem duplication (Figure S3) because both genes are located on chromosome 6, separated by less than 100 kb, and share the same transcriptional orientation—criteria consistent with tandem duplication arising from unequal recombination [36]. In contrast, the four segmental duplication pairs, *StTPP1*/*7*, *StTPP2*/*8*, *StTPP3*/*9*, and *StTPP4*/*5*, were identified based on their positions within large-scale syntenic blocks that reflect ancient whole-genome duplication events in the potato lineage [37] (Figure S3). These duplicated regions exhibit conserved gene order and homology across chromosomes, supporting a segmental origin for these paralogous pairs. All duplicated gene pairs showed Ka/Ks ratios < 1 (Table S2), indicating that they have evolved under purifying selection, which has constrained amino acid changes and likely preserved essential enzymatic functions after duplication. Notably, while *StTPP5* participates in both a segmental duplication (*StTPP4*/*5*) and a tandem duplication (*StTPP5*/*6*), this reflects its involvement in multiple evolutionary events over time. These patterns collectively suggest that the expansion of the TPP family in potato is primarily driven by segmental duplication under stringent functional constraints.

Additionally, synteny mapping anchored each *StTPP* gene to orthologous chromosomal blocks in five reference species (Figure 3). A total of 7, 16, 13, 9, and 8 collinear loci were identified between potato and Arabidopsis, tomato (*Solanum lycopersicum* L., *S. lycopersicum*), eggplant (*Solanum melongena* L., *S. melongena*), pepper (*Capsicum annuum* L., *C. annuum*), and rice, respectively. One-to-one orthology was resolved for *StTPP3* with Arabidopsis *AtTPPH*/*AtTPPI*/*AtTPPJ* and for *StTPP9* with *AtTPPF*/*AtTPPG*, suggesting conserved functional identities. In tomato, three genes (*Solyc04g054930.3.1*, *Solyc04g072920.3.1*, and *Solyc05g051880.3.1*) correspond to *StTPP3*, while three others (*Solyc03g083960.3.1*, *Solyc06g060600.2.1*, and *Solyc08g079060.3.1*) correspond to *StTPP9*. All syntenic pairs exhibited Ka/Ks < 1 (Table S2), indicating persistent purifying selection since lineage divergence. The conserved synteny network thus provides a reliable framework for annotating *StTPP* genes and underscores the evolutionary stability of TPP-mediated regulatory circuits across dicot and monocot lineages.

### 2.5. In Silico Analysis of Cis-Elements in StTPP Promoters

**Figure 4:**
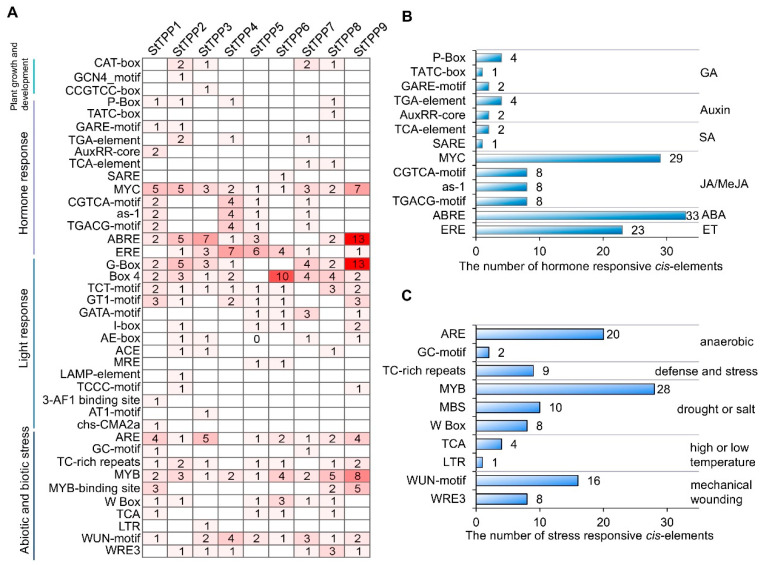
In silico analysis of *cis*-regulatory elements in *StTPP* promoters. (**A**) Distribution of 40 predicted *cis*-acting elements within 2 kb upstream regions of *StTPP* genes, identified using PlantCARE. Elements are categorized into four functional groups: development-, hormone-, light-, and stress-responsive. (**B**) Quantification and classification of hormone-responsive elements across *StTPP* promoters. Bar heights indicate element counts; hormone types are listed on the right. (**C**) Frequency and distribution of stress-responsive elements. Bar heights reflect element numbers, with corresponding stress stimuli annotated on the right.

Bioinformatic analysis of the 2-kilobase (kb) promoter region of each *StTPP* locus revealed a modular architecture of cis-regulatory elements, which can be grouped into four functional clusters (Figure 4). The first cluster comprises developmental regulators, with all promoters containing at least one CAT-box, GCN4-motif, or CCGTCC-box associated with meristem activity, endosperm expression, or vascular development. The second cluster consists of hormone-responsive modules, with every promoter harboring two or more elements responsive to gibberellin, auxin, salicylic acid, jasmonic acid (JA), ABA, or ethylene. JA signaling was the most prevalent, with 53 motifs (primarily MYC-binding sites) distributed across the family, and *StTPP9* containing seven copies. ABA signaling ranked second, represented by 33 ABRE motifs, with 13 in *StTPP9* and seven in *StTPP3* (Figure 4B). The third cluster includes light-responsive elements, with 14 distinct light-associated motifs, including 30 G-box copies in seven promoters. The final cluster contains stress-responsive motifs, with elements linked to anaerobiosis (ARE, GC-motif), defense (TC-rich repeats), drought/salinity (MYB, MBS, W-box), temperature extremes (TCA, LTR), and wounding (WUN-motif, WRE3) (Figure 4C). Collectively, the cis-element repertoire indicates that *StTPP* genes are poised to integrate developmental processes with a broad range of environmental signals in potato.

### 2.6. Gene Regulatory Network of StTPPs

**Figure 5:**
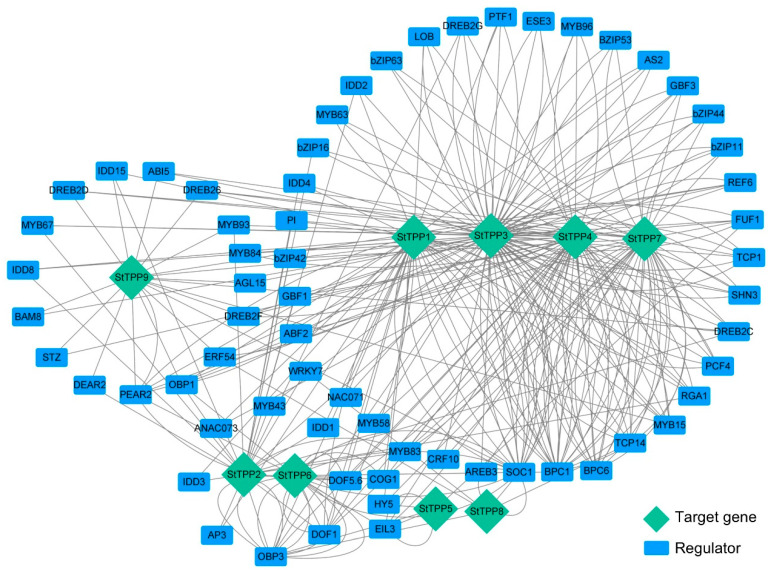
Predicted TF–DNA regulatory network of *StTPP* genes. Promoter regions of individual *StTPP* genes were queried against the PlantRegMap database to identify high-confidence transcription factor (TF) binding sites. The resulting regulatory associations were visualized as an undirected network in Cytoscape 3.10. Blue rectangles represent TFs, green diamonds denote *StTPP* target genes, and gray edges indicate predicted regulatory interactions.

The transcriptional regulation of the *StTPP* genes was mapped using PlantRegMap, with the 2 kb promoter regions as bait, a total of 499 TF-binding sites were assigned to 161 TFs across the nine StTPP loci, with 64 TFs meeting a stringent significance threshold (cutoff *p*-value ≤ 0.05) (Table S3). A gene regulatory network was constructed to visualize the predicted interactions between these 64 TFs and the nine *StTPP* genes (Figure 5). Network topology analysis revealed significant complexity and connectivity, with each StTPP gene receiving input from multiple TFs, including bZIP, MYB, NAC, bHLH, ERF, and WRKY. For instance, 45 TFs are predicted to bind to 88 binding sites on the StTPP3 promoter, highlighting a high degree of combinatorial regulation. This architecture suggests that *StTPP* gene expression is under multifaceted transcriptional control, enabling precise responses to various developmental signals and environmental stimuli.

Several TFs emerged as central regulators, targeting multiple StTPP genes (Figure 5; Table S3). For instance, ABI5, an ABA-responsive TF, is predicted to bind the promoters of *StTPP3*, *StTPP7*, and *StTPP9*, establishing it as a shared node for integrating ABA-mediated stress signals across distinct TPP loci. These hub-like TFs may serve as convergence points for upstream signals, coordinating the transcriptional regulation of functionally related StTPP genes. In contrast, *StTPP5* and *StTPP8* interact with only four or five TFs, indicating more specialized regulation, potentially linked to specific developmental stages or environmental conditions. This network structure emphasizes the redundancy and robustness of TFs in regulating TPP genes and highlights the integration of multiple signaling pathways in modulating Tre6P metabolism.

### 2.7. Tissue-Specific Expression and Subcellular Localization of StTPPs

**Figure 6:**
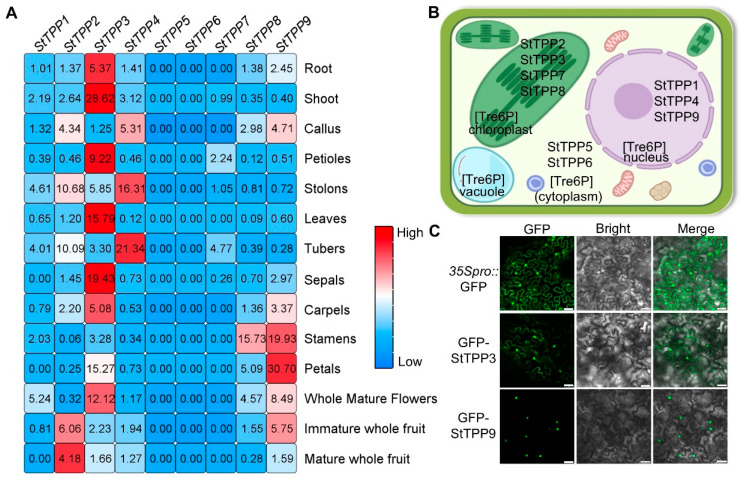
Multi-scale spatial and cellular localization of StTPP expression. (**A**) Spatial transcriptome heatmap of *StTPP* genes derived from RNA-seq data (Spud DB) across 14 tissues: roots, shoots, callus from in vitro plantlets; petioles, stolons, leaves, tubers; floral organs (sepals, carpels, stamens, petals, whole mature flowers); and immature and mature fruits. Transcripts per million (TPM) values were z-score normalized and color-coded: white indicates zero reads, blue represents low expression, and red denotes high expression. Raw data are provided in Table S4-1. (**B**) Subcellular localization of StTPP proteins predicted in silico by WOLF PSORT. (**C**) Subcellular localization of StTPP3 and StTPP9 assessed via Agrobacterium-mediated transient expression in *Nicotiana benthamiana* epidermal cells expressing *35S*::eGFP-StTPP3 or *35S*::eGFP-StTPP9 (green fluorescence). *35S*::eGFP alone served as a control. Fluorescence images were acquired 48 h post-infiltration and merged with bright-field images. Scale bar = 50 µm; images represent three independent experiments.

Spatial expression profiles of the nine StTPP genes were extracted from the Spud DB RNA-seq atlas, which encompasses 14 vegetative and reproductive tissues (Figure 6A). StTPP5, StTPP6, and StTPP7 were either transcriptionally silent or expressed at negligible levels across all samples, suggesting limited involvement in the surveyed organs. Among the expressed genes, StTPP3 exhibited the broadest expression pattern, with high transcript abundance in roots, stems, leaves, flowers, and fruits—indicative of a housekeeping or pleiotropic role. StTPP4 displayed a pronounced bias toward stolon and tuber tissues, with transcript levels exceeding those in other organs by more than fourfold, suggesting a specialized role in subterranean storage organ development. The remaining genes showed moderate or tissue-specific expression patterns—for instance, StTPP1 in whole mature flowers and StTPP8 in stamens. These data indicate that the StTPP family is functionally diversified, comprising both broadly expressed regulators and organ-specific modulators of Tre6P metabolism in potato.

Subcellular localization is a key determinant of protein function. In silico predictions using WoLF PSORT indicated that StTPP proteins may localize to the cytoplasm, nucleus, or chloroplasts, consistent with the multifunctional nature of Tre6P signaling (Figure 6B). To experimentally validate these predictions, we selected StTPP3 and StTPP9 based on two primary criteria: they represent distinct evolutionary lineages—StTPP3 belongs to Clade II and StTPP9 to Clade I; and both exhibit among the highest constitutive expression levels across multiple potato tissues (e.g., leaf, shoot, root, and petal) among all StTPP family members (Figure 6A). GFP fusion constructs under driven by the CaMV *35S* promoter were transiently expressed in *Nicotiana benthamiana* epidermal cells. Confocal microscopy revealed that GFP-StTPP3 localized to both the nucleus and cytoplasm, deviating from initial in silico predictions with WoLF PSORT (Figure 6C). In contrast, GFP-StTPP9 was detected exclusively in the nucleus, aligning with its in silico forecast (Figure 6C). These findings provide critical insights into the subcellular dynamics of StTPP proteins and their potential functional roles in potato physiology.

### 2.8. Validation of StTPPs Differential Expression

**Figure 7:**
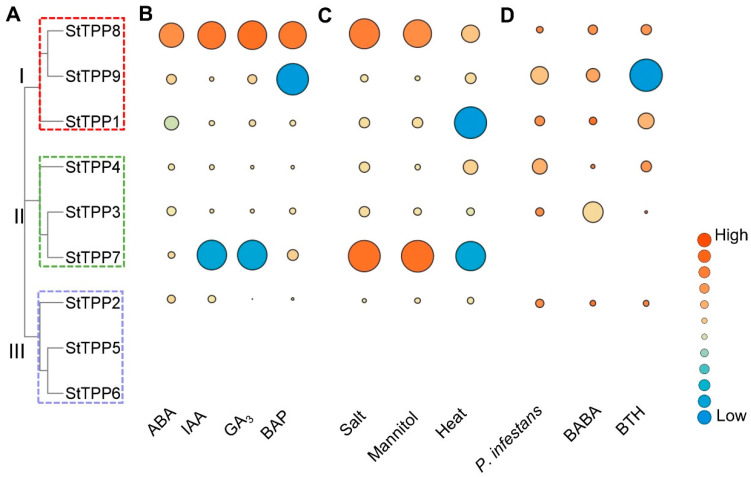
Transcriptional responses of *StTPP* genes under hormonal, abiotic, and biotic stimuli. (**A**) Phylogenetic tree of *StTPP*. (**B**–**D**) Heat-map representation of *StTPP* transcript abundance in whole potato plants after treated with phytohormones (**B**), abiotic stress (**C**), and biotic stress (**D**). Log_2_^FoldChange (treated vs. control)^ is color-coded; red indicates up-regulation, and blue indicates down-regulation.

Integrated transcriptome profiling of the DM potato cultivar revealed that *StTPP* gene expression was precisely and differentially regulated by hormonal, abiotic, and biotic signals. Within the hormone panel, abscisic acid (ABA) strongly suppressed *StTPP1* (log_2_^FoldChange^ = −2.23) while inducing *StTPP8* and *StTPP9* (log_2_^FoldChange^ > 1). Indole-3-acetic acid (IAA) and gibberellic acid (GA_3_) similarly downregulated *StTPP7* and upregulated *StTPP8*, whereas 6-benzylaminopurine (BAP) enhanced *StTPP7* and *StTPP8* expression but repressed *StTPP9*. The consistent upregulation of *StTPP8* across all hormone treatments highlights its central role within multiple signaling pathways (Figure 7B, Table S4-2). Exposure to salt (150 mM NaCl, 24 h) and osmotic stress (260 mM mannitol, 24 h) activated the majority of *StTPP* genes, with *StTPP7* exhibiting the highest fold change. In contrast, heat stress (35 °C, 24 h) elicited a gene-specific response, downregulating *StTPP1* and *StTPP7* while inducing *StTPP4*, *StTPP8*, and *StTPP9* (Figure 7C, Table S4-3). Pathogen-related assays further revealed distinct transcriptional responses. Both *Phytophthora infestans* infection and acibenzolar-S-methyl (BTH) treatment strongly suppressed *StTPP9*, while DL-β-amino-N-butyric acid (BABA) caused the most significant downregulation of *StTPP3* (Figure 7D, Table S4-4).

Collectively, these data indicate that *StTPP* gene expression is precisely and differentially regulated in response to developmental cues, abiotic stresses, and pathogen attacks.

### 2.9. Transcriptional Dynamics of StTPP Genes in Cultivar Xisen6 Under Abiotic Stresses

Our previous studies have established that cultivar Xisen6, a sexual hybrid derived from Shepody and XS9304, sustains robust growth under both in vitro salt stress and the saline soils of the Yellow River Delta [38]. To elucidate the temporal expression dynamics of *StTPP* genes in this salt-tolerant genotype, virus-free plantlets were treated with 80 mM NaCl for two weeks, and samples were collected at 0 h, 3 h, 12 h, and 2 weeks for RT-qPCR analysis of seven *StTPP* members.

**Figure 8:**
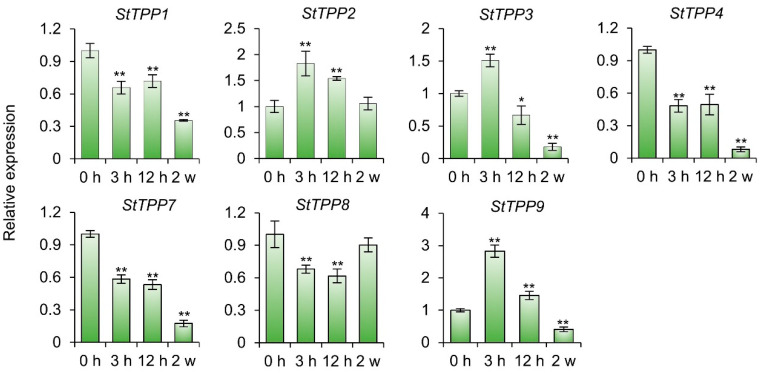
*StTPP* transcriptional dynamics under progressive salt stress. RT-qPCR analysis of *StTPP* expression in potato cultivar Xisen6 treated with 80 mM NaCl for 0 h, 3 h, 12 h, and 2 weeks, respectively. Two-week-old virus-free seedlings subjected to 0 h treatment served as control. Transcript levels were normalized to *StACT* and presented as mean ± SD (n = 3). * *p* < 0.05, ** *p* < 0.01.

As shown in Figure 8, after prolonged salt exposure (2 weeks), transcript levels of five genes were significantly reduced, while *StTPP2* and *StTPP8* remained statistically unaltered. In contrast, early-stage stress (3 h treatment) elicited a pronounced induction of *StTPP2*, *StTPP3*, and *StTPP9* (≥1.5-fold relative to 0 h). The remaining genes exhibited either transient downregulation between 3 and 12 h or consistent suppression throughout the treatment period. These findings suggest that, during the salt response in cultivar Xisen6, specific *StTPP* genes exhibit a biphasic transcriptional response characterized by early activation upon initial salt induction, followed by extensive repression under sustained stress. This suggests that they play distinct roles in both immediate defense and long-term adaptation.

**Figure 9:**
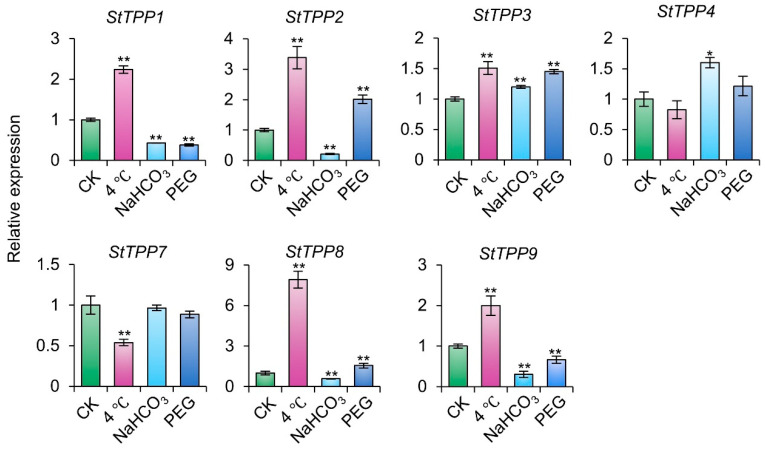
*StTPP* gene expression under distinct abiotic stress conditions. Two-week-old virus-free cultivar Xisen6 potato seedlings were exposed to 4 °C, PEG-6000, or NaHCO_3_ for 12 h, with untreated seedlings as control group (CK). Transcript levels were normalized to *StACT* and presented as mean ± SD (n = 3). * *p* < 0.05, ** *p* < 0.01.

To further characterize the functional responsiveness of *StTPPs* to abiotic stress, transcript abundance was assessed under chilling (4 °C), alkaline (NaHCO_3_), and osmotic (20% PEG) in cultivar Xisen6. As illustrated in Figure 9, each stressor elicited a distinct transcriptional profile across the seven genes. Cold stress led to elevated expression in five *StTPPs*, with *StTPP7* showing a notable reduction. Alkaline treatment induced strong upregulation of *StTPP3* and *StTPP4*, while downregulating four others. Under PEG-induced osmotic stress, *StTPP2* and *StTPP3* were significantly upregulated, whereas three paralogs were suppressed.

Collectively, the *StTPP* gene family exhibits a stress-specific transcriptional landscape, wherein individual members are selectively activated or repressed in response to distinct environmental cues, highlighting their potential roles in adaptive stress responses in potato.

## 3. Discussion

The *TPP* gene family plays a central role in plant growth, development, and stress adaptation by modulating Tre6P levels—a key metabolic signal linking sugar status with cellular responses. These findings collectively position StTPPs as promising molecular targets for engineering abiotic stress resilience in potato without compromising yield potential.

### 3.1. Evolutionary Diversification and Functional Partitioning of StTPPs

In this study, we identified nine *StTPP* genes in potato, forming a compact family phylogenetically structured into three distinct clades (Figure 1B). This number is smaller than those in wheat (14 members) and soybean (23 members), but comparable to Arabidopsis (10 members) and tomato (9 members) [19,24,33,34], suggesting that gene family size is not directly correlated with genome complexity but may reflect functional specialization within lineages. Phylogenetic conservation of clade structure is evident across plants. *StTPPs* cluster with their orthologs in tomato and Arabidopsis, indicating early divergence prior to the Solanaceae–Brassicaceae split. Despite structural conservation of the HAD domain and catalytic motifs I–IV across all *StTPPs* (Figure 2 and Figure S2), functional divergence is evident in gene expression and regulation. The *StTPP* family expanded primarily through segmental duplications, with all paralogous pairs under purifying selection (Ka/Ks < 1; Table S2), suggesting functional constraint rather than neofunctionalization. This evolutionary pattern aligns with potato’s clonal propagation and selective breeding history, where maintenance of core metabolic regulators may be favored over diversification.

Functional roles of TPPs vary across species. In Arabidopsis, AtTPPF, AtTPPI, and AtTPPJ modulate root architecture and stomatal aperture through their enzymatic activity [15,39,40,41], and the *10* × *tpp* multiple knockout exhibits severe developmental defects, including reduced growth, increased shoot branching, and delayed flowering, highlighting the essential role of Tre6P turnover in coordinating source-sink relations and apical dominance [42]. In tomato, *SlTPP3* and *SlTPP4* are transcriptionally activated under drought and salt stress, and their overexpression confers enhanced osmotic stress tolerance without compromising yield [43,44]. In rice, *OsTPP7* is rapidly upregulated during anaerobic germination, reducing Tre6P levels to facilitate starch mobilization [26], while TaTPP-7A in wheat enhances grain filling and yield by modulating the Tre6P–SnRK1 axis and integrating sucrose dynamics with ABA signaling [45]. Notably, some *TPP* homologs have evolved non-catalytic functions. *ZmRA3* in maize regulates meristem size through protein–protein interactions, independent of phosphatase activity [35], implicating RA3 may have multifaceted functions analogous to those of glycolytic enzymes [46], like as sensor and scaffold roles observed in HEXOKINASE1 [47,48]. Collectively, these findings suggest that while TPPs primarily regulate growth, development, and stress responses via enzymatic roles in signaling cascades, certain isoforms may perform non-catalytic signaling functions as moonlighting proteins.

The dual nuclear–cytosolic localization of StTPP3 is consistent with findings in other species. For instance, AtTPPH in Arabidopsis localize to both compartments and are implicated in sugar signaling and stress response coordination [25,49]. In contrast, strictly nuclear-localized isoforms like StTPP9 may function in transcriptional regulation or chromatin-associated processes. These patterns diverge from in silico predictions and support the hypothesis that plant TPPs function as moonlighting proteins, with subcellular localization dictating context-specific Tre6P signaling [25,50]. Given Tre6P’s role as a cytosolic indicator of sucrose status [9,51,52], such spatial compartmentalization may enable localized control of carbon allocation under stress conditions.

### 3.2. Regulatory Complexity Integrates Hormonal and Environmental Cues

Promoter analysis revealed reveals an enrichment of ABRE, MYB, and DREB elements in stress-inducible *StTPP*s (Figure 4), which also found in stress-responsive *TPP*s from rice and maize, but with a higher density of light regulated elements. This may reflect the integration of photoperiodic cues with stress responses in potato, a photoperiod-sensitive tuber, where carbon partitioning must be synchronized with daylength and temperature signals. Network modeling identified 64 high-confidence TFs, predominantly from the bZIP, WRKY, and ERF/DREB families, as candidate upstream regulators of *StTPP* promoters (Figure 5). Experimental evidence in other species corroborates these predictions: DREB1A binds the *AtTPPF* promoter under drought conditions [39], while AaERF64 trans-activates *AaTPPA* during cold acclimation in *Actinidia arguta* [53]. These findings support the role of ERF/DREB-type TFs as evolutionarily conserved regulators of TPP transcription in both eudicots and monocots.

The SnRK1 signaling cascade further contributes to TPP transcriptional control. In rice, OsSnRK1a represses *OsTPP7* expression via phosphorylation of the basic helix–loop–helix TF STARVATION-ASSOCIATED GROWTH INHIBITOR 1 (OsSGI1/OsbHLH111) [54]. In Arabidopsis, SnRK1 phosphorylates bZIP11 to promote *TPP5*/*6* transcription [55,56]. Notably, Tre6P inhibits SnRK1 activity [11,57], establishing a feedback loop that coordinates energy status with transcriptional output. The inferred TF network suggests a similar regulatory mechanism in potato, where SnRK1-dependent phosphorylation of bZIP or DREB factors may modulate StTPP expression in response to metabolic and environmental stimuli. Moreover, MAPK3-mediated stabilization of ICE1 activates *OsTPP1* during cold stress in rice [17], mirroring the induction of *StTPP1*/*2*/*4*/*8*/*9* under chilling conditions in potato (Figure 9). Collectively, these findings delineate a conserved TF–TPP–Tre6P regulatory axis in which stress-responsive and sugar-sensing TFs dynamically regulate TPP transcription to optimize growth, stress resilience, and resource allocation.

### 3.3. Stress-Responsive Transcriptional Dynamics and Physiological Implications

A key distinction lies in expression dynamics during stress. The transcriptional profiling of StTPP genes across diverse hormonal, abiotic, and biotic conditions reveals a highly nuanced and context-dependent regulatory architecture. Notably, *StTPP3* and *StTPP9*, two isoforms with high basal expression and distinct subcellular localizations, are consistently among the most responsive members under early salt stress (3 h) in the tolerant cultivar Xisen6 and are also induced by ABA and osmotic stress in the DM background (Figure 7 and Figure 8). This recurrent responsiveness across genotypes and stress modalities suggests they are sensitive to environmental perturbation.

Furthermore, the observed biphasic pattern, such as early induction followed by repression under prolonged stress, mirrors transient Tre6P dynamics reported in Arabidopsis and maize under salinity [58,59], consistent with a model in which rapid TPP activation may transiently lower Tre6P levels to modulate SnRK1 signaling and redirect carbon flux during stress onset. It must be emphasized that the validity of this model in potato remains speculative. In the absence of physiological, metabolomic, or reverse genetic validation, including undetermined Tre6P, Tre, and downstream metabolic flux in the system, it is currently impossible to fully demonstrate the functional role of *StTPP* members in stress resistance. Nevertheless, the convergence of high expression, stress responsiveness, evolutionary conservation, and subcellular localization makes *StTPP3* and *StTPP9* compelling candidates for future functional characterization.

Collectively, these results position specific *StTPP* isoforms, particularly *StTPP3* and *StTPP9* as high-priority targets for future functional validation. Their integration into the abiotic stress response network appears plausible based on expression kinetics, evolutionary conservation, and parallels in other species, but definitive assignment of physiological roles will require integrated metabolomic, genetic, and phenotypic analyses. Nonetheless, several key questions remain unresolved. The contribution of epigenetic mechanisms to StTPP regulation under stress remains to be elucidated. Additionally, clarification of cell-type–specific TF–StTPP interactions will require high-resolution methods, including tissue-specific promoter systems and single-cell transcriptomic profiling. Furthermore, the possibility that certain StTPPs, such as RA3 [35], fulfill non-enzymatic roles warrants further investigation.

## 4. Conclusions

The *TPP* gene family in potato was comprehensively characterized for the first time in this study. Nine StTPP loci, distributed across five chromosomes, were categorized into three clades based on conserved catalytic motifs and distinct exon-intron structures. Their limited expansion appears to have been shaped by segmental duplication events followed by purifying selection. Promoter analysis identified cis-regulatory elements responsive to hormones, light, and various stresses, with 64 predicted TFs potentially linking *StTPPs* to carbon signaling and environmental responsiveness. Transcriptome profiling in the salt-tolerant cultivar Xisen6 revealed a biphasic expression pattern: early stress elicited transient induction, while prolonged exposure led to broad repression, with stimulus-specific responses under cold, osmotic, and alkaline conditions. Subcellular localization studies showed that StTPP3 is present in both the cytosol and nucleus, suggesting spatially compartmentalized regulation of Tre6P. These results position StTPPs as key modulators of stress adaptation in potato and highlight their potential as candidates for genome editing or marker-assisted selection aimed at enhancing climate resilience. Further investigation into cell-type-specific expression, post-translational regulation, and allelic variation across tetraploid germplasm is warranted to fully harness the Tre6P–TPP pathway in climate-resilient potato breeding.

## 5. Materials and Methods

### 5.1. StTPPs Identification in Potato

To identify members of the *StTPP* gene family in potato (*Solanum tuberosum* L.), protein sequences of AtTPPs retrieved from The Arabidopsis Information Resource (TAIR) (https://www.Arabidopsis.org/, accessed on 30 May 2025) were used as queries in a BLASTP search via the TBtools-II platform against annotated potato protein sequences (DM v6.1) in the Spud DB Potato Genomics Resources database (http://spuddb.uga.edu/, accessed on 30 May 2025) [31,32]. For genes with multiple splice variants, the longest isoform was retained for further analysis. Candidate StTPP proteins were subsequently validated using HMMER’s hmmsearch tool (cutoff = 0.01) (https://www.ebi.ac.uk/Tools/hmmer/search/hmmsearch, accessed on 30 May 2025) [60], as well as through domain confirmation in the NCBI Conserved Domain Database (CDD; http://www.ncbi.nlm.nih.gov/Structure/cdd/wrpsb.cgi, accessed on 30 May 2025) [61] and SMART (http://smart.embl-heidelberg.de, accessed on 30 May 2025) [62].

### 5.2. StTPP Sequence Analysis and Characterization

Physicochemical properties, including amino acid length, molecular weight (MW), theoretical pI, and grand average of hydropathicity (GRAVY), were computed using ExPASy ProtParam (https://web.expasy.org/protparam/, accessed on 30 May 2025) [63]. Predicted subcellular localizations were obtained via WoLF PSORT (https://wolfpsort.hgc.jp/, accessed on 30 May 2025) [64]. Detailed sequences, including protein, CDS, and 2 kb promoter regions of StTPPs, are listed in Table S1.

Conserved motif analysis was performed using the Simple MEME Wrapper in TBtools-II, specifying a maximum of 10 motifs with lengths ranging from 6 to 50 amino acids, allowing for any number of motif repetitions. Gene structures and chromosomal distributions of StTPPs were visualized using the Gene Structure View and Gene Location Visualize functions in TBtools-II [65].

### 5.3. Multiple Sequence Alignment and Phylogenetic Tree Construction

Multiple sequence alignment of full-length *TPP* genes from Arabidopsis, tomato, and potato was conducted using MEGA7 [66] and refined in ESPript 3.0 (https://espript.ibcp.fr/ESPript/cgi-bin/ESPript.cgi, accessed on 30 May 2025) [67]. The protein sequences of tomato SITPP were extracted from the Sol Genomics Network (https://solgenomics.net/, accessed on 30 May 2025). A maximum likelihood phylogenetic tree was generated with 500 bootstrap replicates [68] and visualized using Interactive Tree of Life v6 (iTOL; https://itol.embl.de/, accessed on 30 May 2025) [69].

### 5.4. Collinearity and Repetition Analysis of the StTPPs

The potato, Arabidopsis, and tomato genome sequences were obtained through the database mentioned above, and the genomes of pepper, eggplant and rice were obtained from EnsemblPlants (http://plants.ensembl.org/info/data/ftp/index.html, accessed on 30 May 2025) [70]. Homologous relationships between StTPP genes and TPP genes from *Arabidopsis*, tomato, pepper, eggplant, and rice were analyzed using MCScanX (https://github.com/wyp1125/MCScanX, accessed on 30 May 2025) [71]. Segmental and tandem duplication events within the *StTPP* gene family were identified and visualized using TBtools-II [65]. Evolutionary divergence was assessed by calculating synonymous (Ks) and non-synonymous (Ka) substitution rates of StTPP paralogs with KaKs_Calculator v2.0 (https://sourceforge.net/projects/kakscalculator2/, accessed on 30 May 2025) [72].

### 5.5. Prediction of Cis-Elements and TF-DNA Regulation Networks

The 2 kb upstream promoter regions of *StTPP* genes were retrieved from Spud DB and analyzed for putative *cis*-regulatory elements using PlantCARE (http://bioinformatics.psb.ugent.be/webtools/plantcare/html/, accessed on 30 May 2025) [73].

Predicted TF–*StTPP* regulatory networks were generated via Plant Transcriptional Regulatory Map (http://plantregmap.gao-lab.org/network.php/, accessed on 30 May 2025) [74], which identified homologous *Arabidopsis* TFs potentially binding to *StTPP* promoters. Network visualization was conducted in Cytoscape [75].

### 5.6. Transcriptome Analysis

Transcript abundance of nine *StTPP* loci was retrieved from the Spud DB RNA-seq dataset [31,32]. Transcripts per million (TPM) values were extracted from doubled monoploid (DM) potato sampled across vegetative and reproductive organs. For abiotic assays, vitro plantlets were subjected to 150 mM NaCl, 260 mM mannitol, or 35 °C heat for 24 h. Parallel hormone treatments were performed with 50 µM ABA, 10 µM IAA, or 50 µM GA_3_ under identical conditions. Biotic challenges included inoculation with *Phytophthora infestans* (*P. infestans*) and elicitation with 100 mg/mL acibenzolar-S-methyl (BTH), and DL-β-amino-N-butyric acid (BABA); leaf material was harvested at 24, 48 and 72 h post-treatment. Accession identifiers for every transcriptomic data are cataloged in Table S5.

### 5.7. Plant Materials and Growth Conditions

Sterile single-node cuttings of *Solanum tuberosum* cultivar Xisen6 provided by the National Potato Engineering Research Center/Laoling Xisen Potato Industry Group Co., Ltd. (Laoling, Shandong, China) were propagated on half-strength Murashige and Skoog (1/2 MS) medium supplemented with 1% (*w*/*v*) sucrose and 0.8% agar and maintained at 25 °C under a 16 h light/8 h dark photoperiod (100 µmol m^−2^ s^−1^). Two-week-old seedlings grown in liquid 1/2 MS were exposed to abiotic stress treatments as follows: (i) salt stress: 80 mM NaCl (pH 5.8); (ii) alkali stress: 50 mM NaHCO_3_ (pH 7.2); (iii) drought stress: 20% (*w*/*v*) PEG-6000 (pH 5.8); and (iv) cold stress: 4 °C exposure. Control seedlings were maintained in untreated liquid 1/2 MS medium (pH 5.8). Whole seedlings were harvested at designated time points, flash-frozen in liquid nitrogen, and stored at −80 °C for RNA extraction. Each treatment included three biological replicates.

### 5.8. Total RNA Isolation and Expression Analysis

Total RNA was extracted from liquid nitrogen-ground seedlings using the Plant Total RNA Extraction Kit (AC0305, Sparkjade, Jinan, China). One microgram of total RNA per sample was reverse transcribed with the All-in-One First-Strand Synthesis MasterMix Kit (EG15133S; Yugong Biolabs, Lianyungang, China). RT-qPCR was performed according to previously established protocols [76]. Potato *Actin* (*StACT*, accession number: Soltu.DM.11G008990) was used as the reference gene to normalize transcript levels across samples. Primer sequences are listed in Table S6.

### 5.9. Subcellular Localization Analysis of StTPP3 and StTPP9

Full-length coding sequences (excluding stop codons) of *StTPP3* and *StTPP9* were amplified with gene-specific primers (Table S6) and cloned into the linearized pCambia1300-eGFP vector using seamless recombination (One Step Cloning Kit, 10911ES50, Yeasen, Shanghai, China). The resulting *35S*:eGFP-StTPP fusion constructs and the *35S*:eGFP empty vector were introduced into *Agrobacterium tumefaciens GV3101* and transiently expressed in *Nicotiana benthamiana* leaves. After 48 h incubation at 23 °C, eGFP fluorescence was imaged via confocal microscopy (SP5II; Leica, Wetzlar, Germany).

### 5.10. Statistical Analysis

All experiments were conducted in triplicate. Data are expressed as mean ± standard deviation (SD). Statistical significance was assessed using Student’s *t*-test in Excel 2021 (Microsoft Corp., Redmond, WA, USA); * *p* < 0.05 was considered significant, and ** *p* < 0.01 was considered highly significant.

## Figures and Tables

**Table 1:** Identified *StTPP* family members in the potato genome.

Gene Name	Locus ID (V6.1)	Number of Amino Acid (aa)	Protein MW (Kda)	Theoretical pI	GRAVY
*StTPP1*	Soltu.DM.03G015270	384	42.99	7.60	−0.342
*StTPP2*	Soltu.DM.03G022770	341	38.34	5.44	−0.311
*StTPP3*	Soltu.DM.04G021620	386	43.48	9.4	−0.382
*StTPP4*	Soltu.DM.04G029210	365	41.39	9.45	−0.512
*StTPP5*	Soltu.DM.04G037760	289	32.84	5.73	−0.194
*StTPP6*	Soltu.DM.04G037770	293	33.09	5.49	−0.228
*StTPP7*	Soltu.DM.05G021910	375	42.78	9.44	−0.422
*StTPP8*	Soltu.DM.06G017740	339	38.43	6.47	−0.431
*StTPP9*	Soltu.DM.08G025340	384	43.47	8.61	−0.423

## Data Availability

All data supporting the findings of this study are included in the manuscript and its supplementary information files. All databases used in this study are open for public and the links are as follows: Spud DB: http://spuddb.uga.edu/; Sol Genomics Network: https://solgenomics.net/; TAIR: https://www.arabidopsis.org/; Ensembl plants: http://plants.ensembl.org/info/data/ftp/index.html; Pfam: http://pfam.xfam.org; CDD: http://www.ncbi.nlm.nih.gov/Structure/cdd/wrpsb.cgi; SMART: http://smart.embl-heidelberg.de; ExPASy: https://web.expasy.org/protparam/; WoLF PSORT: https://wolfpsort.hgc.jp/; KaKs_Calculator v2.0: https://sourceforge.net/projects/kakscalculator2/; MCScanX: https://github.com/wyp1125/MCScanX; iTOL: https://itol.embl.de/; ESPript 3.0 https://espript.ibcp.fr/ESPript/cgi-bin/ESPript.cgi; MEME: http://meme.nbcr.net/meme/ (accessed on 30 May 2025); PlantCare: http://bioinformatics.psb.ugent.be/webtools/plantcare/html/; Plant Transcriptional Regulatory Map: http://plantregmap.gao-lab.org/network.php.

## Supplementary Materials

The following supporting information can be downloaded at https://www.mdpi.com/article/10.3390/plants14213300/s1, Figure S1. Motif sequences identified in StTPP proteins. Figure S2. Multiple sequence alignment of the TPP proteins from potato, Arabidopsis, tomato and RA3 from maize. Figure S3. Collinearity analysis of *TPP* gene family in potato. Table S1. StTPPs sequence information. Table S2. The value of Ka_Ks in syntenic analysis. Table S3. Transcription factors regulation prediction results. Table S4. Transcriptome data of *StTPP* genes expression pattern from Spud DB. Table S5. Accession numbers of transcriptomic data from Spud DB. Table S6. Primers used in this experiment.
