# Supplementary material for: Genome-Wide Analysis of Trehalose-6-Phosphate Phosphatases (TPP) Gene Family in Potato (Solanum tuberosum) Reveals Functional Divergence Under Stress
Source: Plants (Basel). 2025 Oct 29;14(21):3300. doi: 10.3390/plants14213300 (PMC12608325; doi:10.3390/plants14213300)
Supplement: Supplementary file 1 [file plants-14-03300-s001.zip › Supplemental figure legends.pdf]

Supplemental figure legends

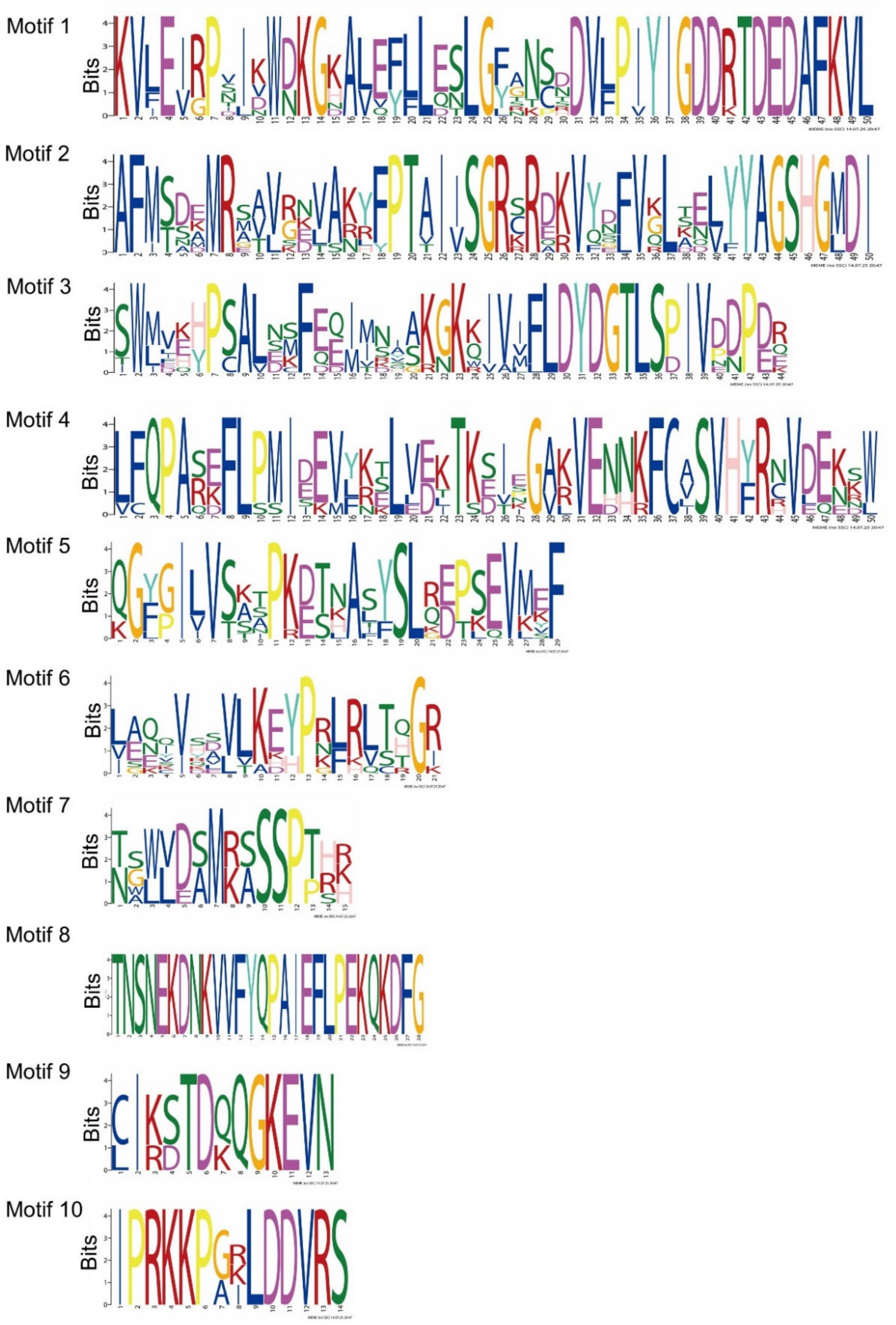

Figure S1. Motif sequences identified in StTPP proteins.

Motif discovery was conducted using MEME. The height of each amino acid reflects its conservation frequency. The scale bar below represents motif sequence length.

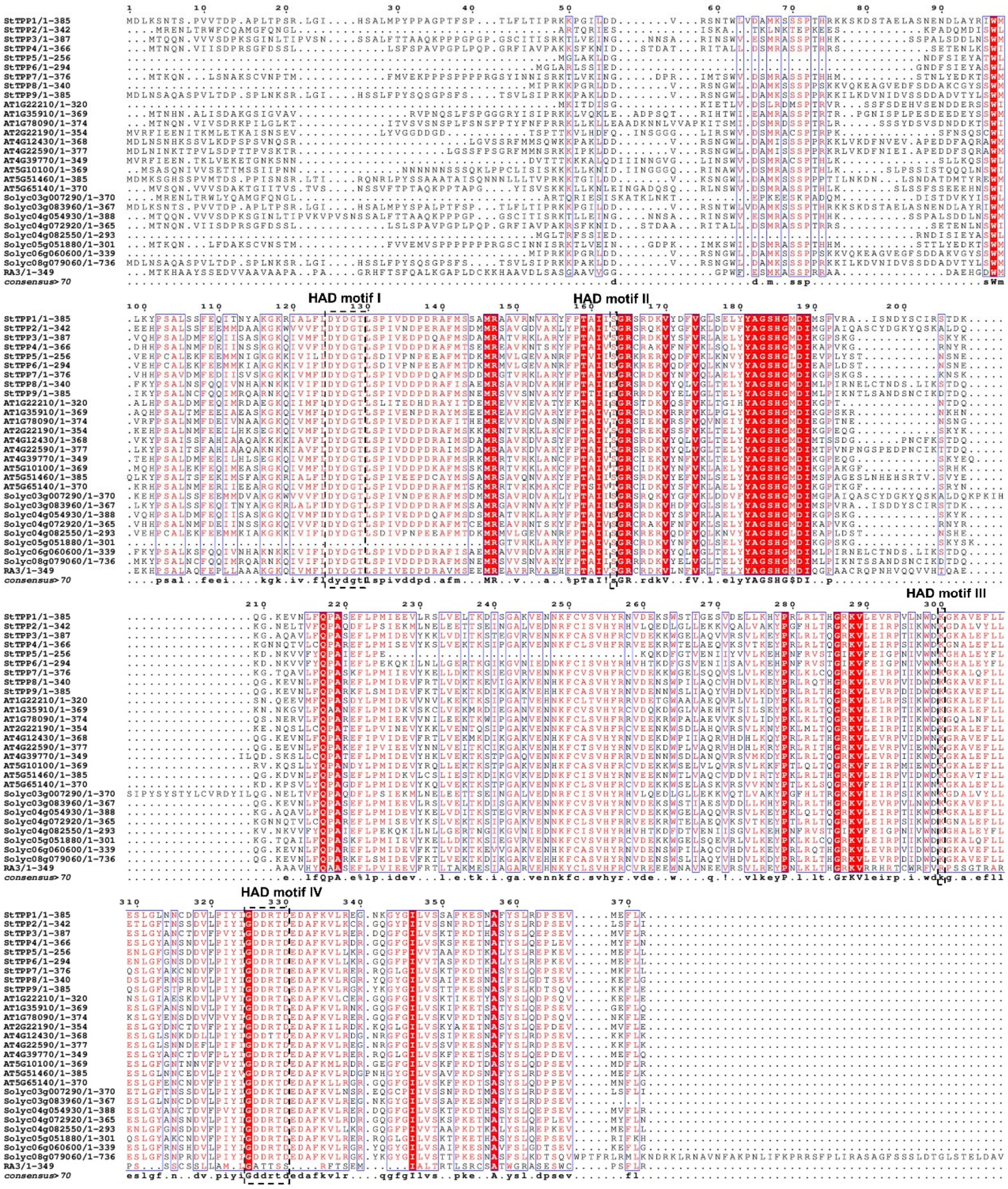

Figure S2. Multiple sequence alignment of the TPP proteins from potato, Arabidopsis, tomato and RA3 from maize.

TPP protein sequences from potato, Arabidopsis, tomato and RA3 in maize, Boxes highlight the multiple sequence alignment containing the conserved motifs I－IV of the HAD superfamily, which define the TPP catalytic domain. Alignment performed using MEGA7 and visualized in ESPript 3.0.

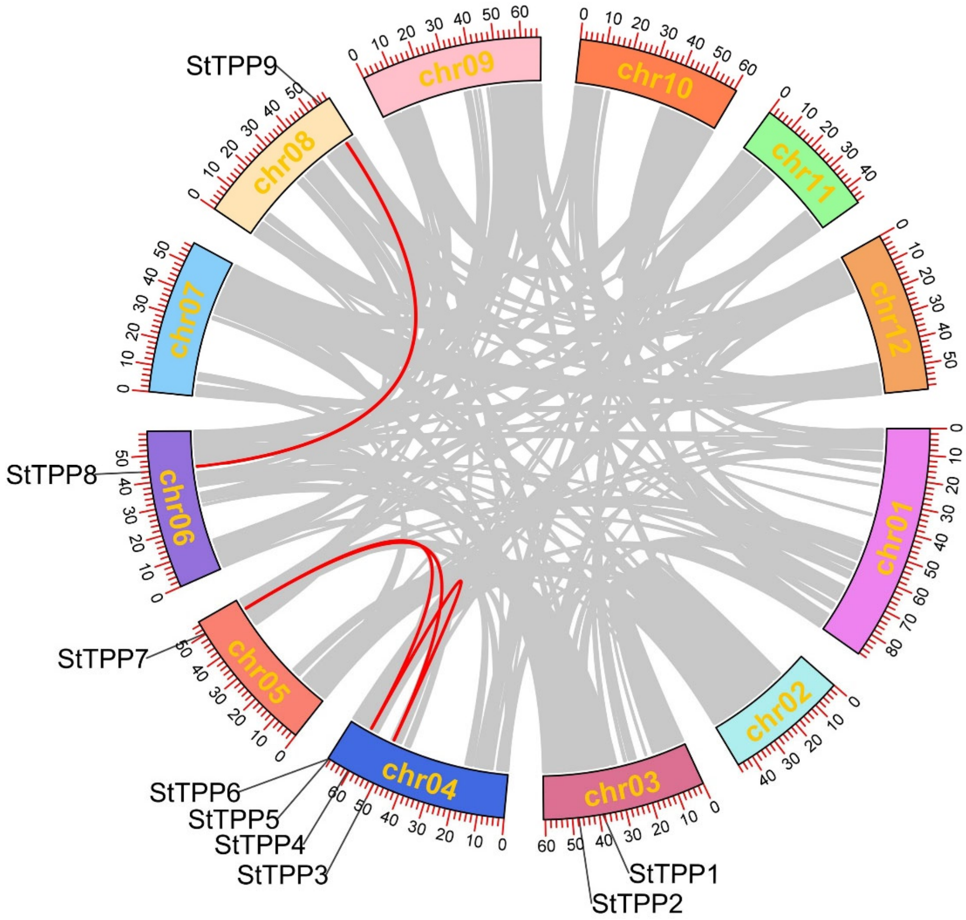

Figure S3. Collinearity analysis of the TPP gene family in potato.

MCScanX illustrated segmental duplications among StTPP loci. Chromosome numbers appear inside each circle; StTPP gene positions are indicated outside. Red arcs denote segmentally duplicated gene pairs.
