# Supplementary figures and images for: Genome-Wide Analysis of Trehalose-6-Phosphate Phosphatases (TPP) Gene Family in Potato (Solanum tuberosum) Reveals Functional Divergence Under Stress
Source: Plants (Basel). 2025 Oct 29;14(21):3300. doi: 10.3390/plants14213300 (PMC12608325; doi:10.3390/plants14213300)

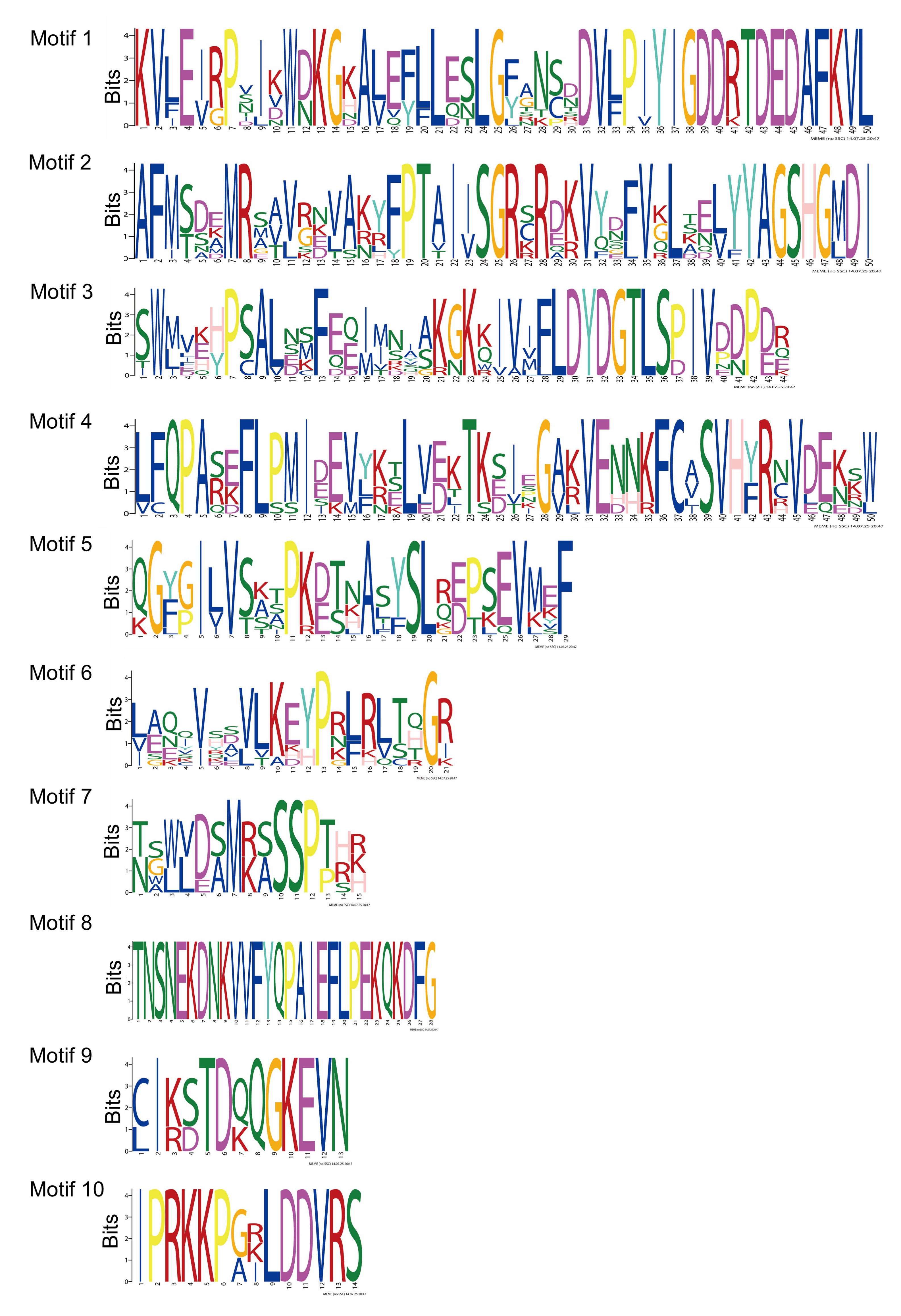

Supplement: Supplementary file 1 [file plants-14-03300-s001.zip › Figure S1.jpg]

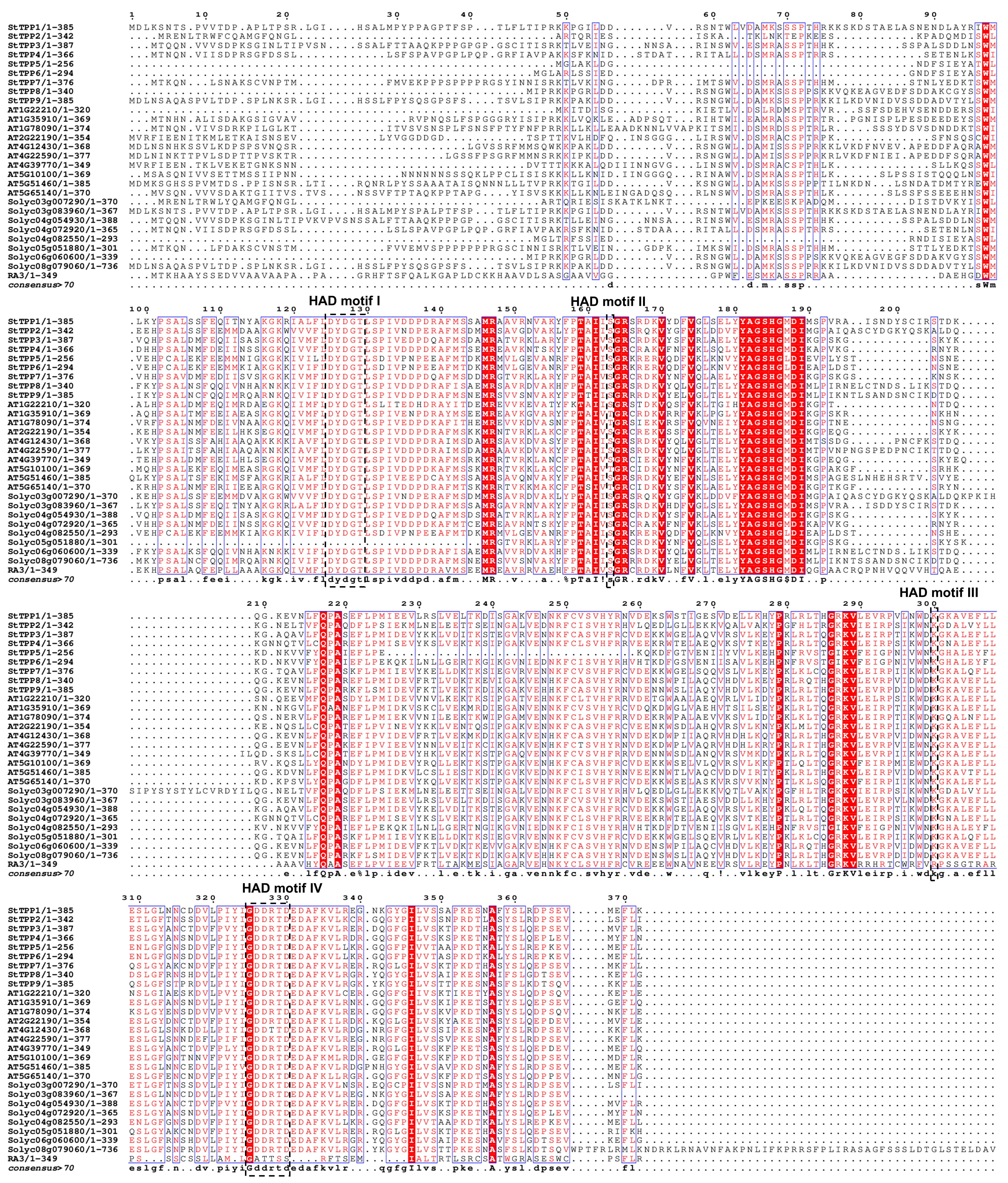

Supplement: Supplementary file 1 [file plants-14-03300-s001.zip › Figure S2.jpg]

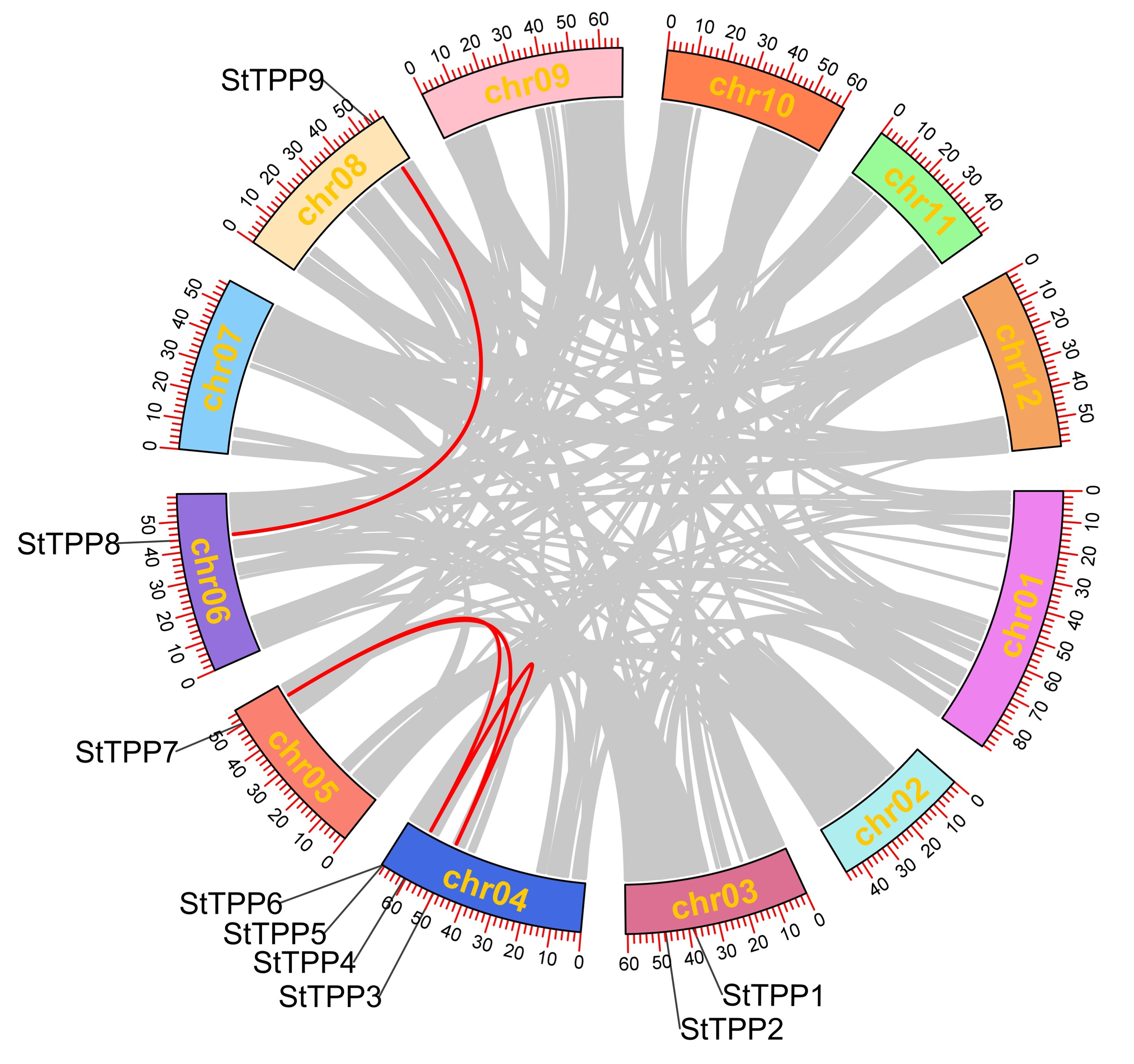

Supplement: Supplementary file 1 [file plants-14-03300-s001.zip › Figure S3.jpg]
